# Rumination in bereaved parents: Psychometric evaluation of the Swedish version of the Utrecht Grief Rumination Scale (UGRS)

**DOI:** 10.1371/journal.pone.0213152

**Published:** 2019-03-19

**Authors:** Josefin Sveen, Lilian Pohlkamp, Ulrika Kreicbergs, Maarten C. Eisma

**Affiliations:** 1 Department of Health Care Sciences, Palliative Research Centre, Ersta Sköndal Bräcke University College, Stockholm, Sweden; 2 National Center for Disaster Psychiatry, Department of Neuroscience, Uppsala University, Uppsala, Sweden; 3 Department of Women’s and Child’s Health, Karolinska Institutet, Stockholm, Sweden; 4 Department of Clinical Psychology and Experimental Psychopathology, University of Groningen, Groningen, Netherlands; Universitat d'Alacante, SPAIN

## Abstract

**Background:**

Bereaved parents may be at higher risk to develop persistent, severe and disabling grief, termed prolonged grief. Grief rumination, repetitive thinking about the causes and consequences of the loss, is a malleable cognitive process that maintains prolonged grief. Grief rumination can be measured with the Utrecht Grief Rumination Scale (UGRS). The present study aimed to examine the psychometric properties of the new Swedish version of the UGRS in a sample of bereaved parents.

**Methods:**

A Swedish nationwide postal survey including measures of demographic and loss-related variables, grief rumination (UGRS), and symptoms of prolonged grief, posttraumatic stress, anxiety, depression, and insomnia, was completed by 226 parents (133 mothers and 93 fathers) who lost a child to cancer in the past five years. Psychometric properties of the UGRS were examined through confirmatory factor analyses (CFA), reliability analyses, and assessment of UGRS score associations with symptoms of prolonged grief, posttraumatic stress, depression, anxiety, and insomnia.

**Results:**

The internal consistency of the Swedish UGRS was good. The CFA yielded an acceptable fit for a two-factor hierarchical model with five sub-factors. Grief rumination was positively associated with all psychopathology symptom measures. Higher scores on UGRS were found in parents with possible prolonged grief disorder compared to those without (*d* = 1.47). Moreover, the Swedish UGRS was associated with prolonged grief symptoms over and above loss-related and demographic variables and other psychopathology symptoms.

**Conclusions:**

The Swedish UGRS demonstrated good psychometric properties, which supports its use as a measure to assess grief rumination in Swedish bereaved parents in research and practice.

## Introduction

Despite the fact that most people adjust to bereavement without professional intervention, this life-event is associated with an increased risk of developing a wide range of physical and mental health problems [1, 2] and increased mortality [3]. Some subgroups of bereaved people may be at a heightened risk to develop mental health problems, including people who experience violent loss or the loss of a partner or child [4, 5].

A significant minority of bereaved people experiences persistent and severe grief reactions, commonly termed complicated grief or prolonged grief [6, 7]. Throughout this manuscript we will use the term prolonged grief to refer to such grief reactions. The latest proposal to define a disorder characterised by prolonged grief, is prolonged grief disorder (PGD), which will be included in the WHO International Classification of Diseases’ 11^th^ revision (ICD-11) [8]. PGD is characterized by intense and persistent longing for the deceased and/or cognitive preoccupation with the deceased and additional symptoms indicative of emotional distress including emotions such as guilt, anger, and sadness, and difficulty accepting the death [9]. These reactions should persist at least six months after the death and be accompanied by functional impairment [8]. Bereaved parents may be among the most vulnerable to develop prolonged grief [10–12]. For example, two recent studies report prevalence rates for prolonged grief 13% and 16%, respectively, in parents who lost a child to cancer [10, 13], whereas a meta-analysis showed an estimated pooled prevalence rate of prolonged grief following natural losses of 9.8% (95% CI 6.8–14.0) [14].

The changing status of prolonged grief in diagnostic handbooks such as the ICD-11 make it increasingly important to better understand the potential mechanisms underlying disturbed grief responses. Understanding modifiable cognitive processes that play a role in the development and maintenance of prolonged grief can help in determining what techniques may prove most effective in the treatment of PGD [15]. One such cognitive process is rumination, broadly defined as the process of thinking repetitively and/or recurrently about the causes and consequences of negative events and/or negative emotions [16]. Historically, two types of rumination have been extensively studied in adjustment to bereavement: depressive rumination and grief rumination [17, 18].

Depressive rumination is defined as repetitively and passively focusing on depressive symptoms and the causes and consequences of these symptoms [17]. Depressive rumination is associated with higher levels of anxiety, depression, prolonged grief and posttraumatic stress and general distress following loss [18]. An often-used theory to understand the maladaptive effects of depressive rumination is the Response Styles Theory (RST) [17]. The RST holds that rumination leads to poorer adjustment to bereavement because people repeatedly focus on their negative emotions which prolongs loss-related distress. Specifically, rumination is proposed to increase negative mood by enhancing the accessibility of negative thought content. This, in turn, makes problem solving less effective as people have more negative thoughts about the effectiveness of their problem solutions and become less likely to implement such solutions. Additionally, repeatedly going over ruminative thoughts with friends and family may drive away social support, further exacerbating distress [17, 19].

Grief rumination, on the other hand, has been defined as repetitive and recurrent thinking about causes and consequences of the loss and negative loss-related emotions [15, 18, 20–22]. Grief rumination includes counterfactual thinking about the loss (i.e. imagining alternative realities in which the person would not have died), thinking about the unfairness of the loss, the meaning and consequences of the loss, analyzing one’s own emotional reactions to the loss, and others’ responses to the loss [23]. Stroebe and colleagues’ Rumination as Avoidance Hypothesis (RAH) suggests that rumination is maladaptive because chronically high levels of grief rumination serve as a cognitive avoidance strategy [24]. For example, a person may repeatedly rehash what could have been done to prevent the loss in order to avoid being confronted with the thoughts about the permanence of the separation from the deceased. This may have negative consequences as it hinders acceptance of the loss and/or the integration of memories about the loss into the existing autobiographical memory base, thereby maintaining prolonged grief [25]. Evidence for this position has accumulated over the past years, with multiple surveys, experiments and treatment trials supporting a unique link between rumination and cognitive, emotional and behavioral avoidance strategies following bereavement [18].

While some subtypes of grief rumination were already studied in late eighties [26, 27], the research body on grief rumination has grown relatively rapidly over the past two decades. The results of this research demonstrate that grief rumination is concurrently and/or longitudinally positively associated with symptoms levels of depression, anxiety, posttraumatic stress and prolonged grief [18, 20–22, 28]. Furthermore, grief rumination has been shown to be a better concurrent and longitudinal predictor of depressive and prolonged grief symptoms than depressive rumination [23]. Grief rumination therefore appears more relevant in explaining negative mental health outcomes following bereavement than depressive rumination. Whilst not previously studied in the context of bereavement, it can be expected that the well-established association between rumination and sleep problems (for a review: [29]), may also be present in bereaved people. Establishing such associations appears important given the growing evidence that sleep disturbances are interlinked with mental health problems following loss, including prolonged grief [30].

Recently, Eisma and colleagues developed and validated a first standardized instrument to assess grief rumination, the Dutch Utrecht Grief Rumination Scale (UGRS) [31]. Thirty items were constructed based on theories and expert opinions on depressive rumination, trauma-related rumination and grief-relevant rumination. The 30-item scale was then administered in a sample of bereaved individuals. A five-factor structure was found and the three highest loading items from each factor were retained. Next, a second factor analysis showed that the remaining 15 items also loaded high on a single factor, suggesting that the UGRS has a hierarchical factor structure. Together, this appears to support the idea that subtypes of rumination can be studied independently, yet together assess the overarching construct of grief rumination. The Dutch version assess the aforementioned subtypes of grief rumination (i.e., counterfactual thinking, thinking about the loss’ unfairness, meaning and consequences, one’s own emotional reactions to the loss, and others’ responses to the loss) [31] and has been translated and validated in English [23]. In confirmatory factor analyses a single-level factor structure with five factors provided the best model fit, while a hierarchical model with a second order factor performed almost equally well to UGRS data in Dutch and English bereaved samples [23]. The UGRS has demonstrated excellent internal consistency and evidence was provided for its convergent, divergent, discriminant and concurrent validity [23, 31]. Two recent validations studies, a German version of the UGRS [32] and a Chinese version of the UGRS [33] both demonstrate comparable psychometric properties to the Dutch and English scale.

Given the clinical and theoretical relevance of rumination, more international research is needed to enhance the understanding of grief rumination in adjustment to bereavement, in particular for groups at risk to develop prolonged grief. Therefore, the present study aimed to assess the psychometric properties of the Swedish version of the UGRS in a sample of cancer-bereaved parents. Reliability was assessed by examining the internal consistency of the UGRS. Construct validity of the UGRS was examined with confirmatory factor analysis (CFA) and through assessment of the inter-correlations among UGRS subscales. Further, discriminant validity was investigated by comparing UGRS scores between parents with and without possible PGD. Concurrent validity was investigated by examining associations between grief rumination and measures of prolonged grief, posttraumatic stress, depression, anxiety and insomnia symptoms. Moreover, a hierarchical regression was conducted, to test the hypothesis that rumination will show a positive association with PGD symptoms over and above relevant demographic and loss-related variables and all other psychopathology symptoms.

## Materials and methods

### Procedure and participants

This psychometric study of the UGRS was based on data from a Swedish nationwide postal survey. By linking the Swedish Cause of Death Registry with the Swedish Childhood Cancer Registry 530 children diagnosed with a malignancy before the age of 17 and who died due to the malignancy before the age of 25 in the period from 2010 to 2015 were identified. Next, the children’s parents were identified through the Swedish Population Registry. Inclusion criteria for the parents were a good understanding of the Swedish language and living in Sweden at the time that the study was conducted. Five-hundred and twelve parents met the inclusion criteria and were sent an information letter describing the purpose and procedure of the study. The letter included contact details of a researcher who could be approached if the parents had any further questions. The mother and father of each child were contacted separately by telephone about one week later to ask for their verbal consent to participate in the study. Some parents e-mailed the research group that they wanted to take part in the study after they received the letter, hence they were not contacted by telephone. The consent responses were noted and saved on a secure server. Of the eligible participants, 76 parents could not be reached, 63 parents declined participation, and 373 consented and were sent a questionnaire with a prepaid envelope to return the questionnaire. Participants received a reminder telephone call if the questionnaire was not returned within one month. Of the 373 parents who consented to participate, 232 (62%) returned the questionnaire. There were no statistical difference between responders (*n* = 232) and non-responders (*n* = 280) on the parents’ age at time of study, t(510) = 0.57, p = .57, the parent’s age when child died, t(431) = 0.43, p = .67, years since loss, t(510) = 0.58, p = .56, the deceased child’s age at diagnosis, t(510) = 1.35, p = .18, and at death, t(510) = 0.95, p = .35) and the child’s gender, χ^2^ (1) = 0.02, p = .93. However, there were more mothers than fathers in the group that responded compared to the group that did not respond, χ^2^ = (1) 8.83, *p* = .002. The sample included in the present study consisted of 226 parents who had completed all items of the UGRS. The study (including the consent procedure) was approved by the Regional Ethical Review Board in Stockholm, Sweden (Reg. No. 2015/2183-31/5).

### Measures

#### Demographic and loss-related variables

Information on the child’s type of cancer diagnosis, gender, age at the time of diagnosis, and time of death, and time between diagnosis and death were obtained from the Childhood Cancer registry. Information on parents’ age, gender, marital status, education level, number of siblings, and if the child had a relapse or relapses were obtained from the survey using a self-constructed questionnaire.

#### Grief rumination

The Utrecht Grief Rumination Scale (UGRS) [23, 31] contains 15 items that measure different aspects of grief rumination, defined as thinking repetitively and recurrently about causes and consequences of a loss and loss-related emotions. Participants are asked to rate how often they have experienced certain thoughts over the past month on a five-point scale ranging from 1 (never) to 5 (very often). Total scores range from 15 to 75 and generate an overall grief rumination score. The scale can be divided to five subscales: *Meaning* (items 1, 2, 15) assesses thoughts about the meaning and the consequences of the death; *Relationships* (items 3, 9, 14) measures thoughts related to reactions from others; *Counterfactuals* (items 4, 8, 10) assess counterfactual thinking about the events leading to the loss; *Injustice* (items 5, 11, 12) captures thoughts about the injustice of the loss; *Reactions* (items 6, 7, 13) assess how analyses of one’s emotional reactions to the loss. The UGRS items are cited in a previous study [23]. The UGRS was translated into Swedish by two researchers (JS & LP) independently at Palliative Research Centre, Ersta Sköndal Bräcke University College, Stockholm in 2015 and subsequently back-translated by a professional translator and approved by the original author. The Swedish version can be obtained from the authors.

#### Symptoms of prolonged grief

The Prolonged Grief Disorder-13 (PG-13) was used to assess prolonged grief symptoms, according to Prigerson’s 2009 criteria (which differ from recently released ICD-11 criteria) [7]. The PG-13 instrument contains 11 items assessing cognitive, behavioral and emotional symptoms and are used in this study. PG-13 also contains one duration item and one impairment item. The first four symptom items are rated on a frequency scale from 1 (not at all) to 5 (several times a day), and items 6–12 are rated on an intensity scale from 1 (not at all) to 5 (overwhelmingly), yielding a total score range between 11 and 55. The duration and impairment items are dichotomous items (Yes/No). A recent study of the Swedish PG-13 have shown satisfactory psychometric properties and a preliminary cutoff score of ≥ 35 indicating possible PGD [13]. In this study, the PG-13 showed good internal consistency, α = .89.

#### Symptoms of posttraumatic stress

The PTSD Checklist for DSM-5 (PCL-5) was used to measure symptoms of posttraumatic stress disorder (PTSD) [34]. It contains 20 items corresponding to the symptoms of PTSD in the DSM-5, and the items are rated on a scale from 0 (not at all) to 4 (extremely). Total scores range from 0 to 80 and a cutoff score of 33 is recommended as a clinical cut-off for a probable PTSD diagnosis. A preliminary validation study of the Swedish PCL-5 has shown adequate psychometric properties [35]. In the present sample, the PCL-5 demonstrated excellent internal consistency, α = .95.

#### Symptoms of anxiety

The Generalized Anxiety Disorder-7 (GAD-7) was used to assess self-reported generalized anxiety disorder symptoms [36, 37]. It consists of 7 items, rated on a scale from 0 (not at all) to 3 (nearly every day), with a total score range between 0 and 21. In the current study, the internal consistency of the GAD-7 was excellent, α = .92.

#### Symptoms of depression

The Montgomery-Åsberg Depression Rating Scale (MADRS) [38] was used to assess depressive symptoms during the past three days. It contains 9 items rated on a scale from 0 to 6 and the total score ranges between 0 and 54, where higher scores indicate more depressive symptoms. In the present study, the internal consistency of the MADRS was excellent, α = .90.

#### Symptoms of insomnia

The Insomnia Severity Index (ISI) [39] was used to measure self-reported insomnia severity. It consists of 7 items, partly corresponding to the symptoms in DSM-5, assessing the severity of sleep-onset and sleep maintenance difficulties, sleep pattern satisfaction, interference with daily functioning, noticeability of impairment attributed to the sleep problem, and degree of concern or distress caused by the sleep problem. The items are rated on a scale ranging between 0 and 4 and the total score ranges from 0 to 28. In the current sample, the internal consistency was excellent, α = 94.

### Statistical analyses

The data analysis was performed with IBM SPSS statistics 22. Prior to the main analyses of correlations, t-test and linear regression, non-violation of assumptions were checked for all relevant variables, including assessment of outliers, homogeneity of variance and linearity, multicollinearity, independent errors, and normal distribution of errors. The reliability of the UGRS was investigated by evaluating the internal consistency using corrected item-total correlations (with the item itself excluded from the sum score), Cronbach’s α coefficients and estimations of α when one item is omitted, Omega coefficients, and mean inter-item correlations. As Omega coefficients yielded highly similar results to the Cronbach’s α coefficients only the latter are reported. A Cronbach’s α > .70 was regarded as satisfactory [40], whereas the recommended range of mean inter-item correlations is .15 - .50 [41]. The mean inter-item correlations are not affected by the number of items as opposed to α and thus provides additional reliability information.

Confirmatory factor analyses were conducted via Mplus 8 [42] using robust weighted least-squares with a mean and variance adjustment (WLSMV) estimator. While prior studies on the UGRS used ML estimation [23, 31, 32], WLSMV method is strongly recommended for data with ordinal items, which single items from Likert scales are argued to be [43–45]. Based on previous studies [23, 32], two models were tested, namely a five-factor correlated model (which would indicate that the subtypes of grief rumination are related) and a second-order five-factor model in which the five subscales are themselves the indicators for one higher-order factor (which would indicate that subtypes of grief rumination are related, yet also represent a higher-order construct of grief rumination). Goodness of model fit was assessed with the χ2 test, the comparative fit index (CFI) and the Tucker-Lewis Index (TLI), the root mean square error of approximation (RMSEA), and Weighted root-mean-square residual (WRMR). The following standards were used in assessing model fit: a non-significant test of model χ2, χ2/df of between 2 and 3, CFI and TLI > .90, RMSEA < .08 [46], and WRMR < 1.0 [47, 48]. Discriminant validity, which is a type of construct validity, was investigated by comparing UGRS scores between individuals with possible PGD with individuals without PGD. Participants with a PG-13 score ≥ 35 [13] was defined as having possible PGD. Cohen’s *d* was reported as a measure of effect size. To examine concurrent validity Pearson correlations were computed between rumination and symptoms of prolonged grief, posttraumatic stress, depression, anxiety and insomnia as well as sociodemographic and loss-related variables. To compare correlations coefficients Fisher’s *z*-tests were conducted. In addition, a hierarchical multiple regression with prolonged grief symptoms as the dependent variable was conducted to test the concurrent validity of the Swedish UGRS. Using blockwise entry, sociodemographic and loss-related variables related to prolonged grief symptoms were entered first. The second step additionally included symptoms of posttraumatic stress, depression, anxiety and insomnia. In the last step, the UGRS total score was entered in the model.

## Results

### Sample characteristics

Participants had a mean age of 46.22 years (*SD* = 8.32). The sample contained 133 mothers (59%) and 93 fathers (41%) of 152 deceased children, including 74 parent dyads. On average, time since loss was 3.85 years ago (*SD* = 1.44). The most prevalent types of cancer were brain tumor, 39% (*n* = 59), and leukemia, 28% (*n* = 42). The mean age of the children was at time of diagnosis 7.28 years (*SD* = 5.32) and at time of death 10.02 years (*SD* = 6.62). Of the deceased children 44% (*n* = 67) were girls and 56% (*n* = 85) were boys.

### Preliminary analyses

Prior to the main analyses the assumptions for conducting correlations, t-test and linear regressions were checked. First, an analysis of standard residuals was carried out, which showed that the variables contained no outliers (Std. Residual Min = -3.24, Std. Residual Max = 2.30). Tolerance and VIF factors were inspected and these indicated that multicollinearity was not a concern. The data met the assumption of independent errors (Durbin-Watson value = 2.01). The histogram of standardised residuals and the normal P-P plot indicated that the data contained approximately normally distributed errors. The scatterplot of standardised residuals showed that the data met the assumptions of homogeneity of variance and linearity.

### Reliability

The internal consistency of the total UGRS was excellent, α = .92, and it did not improve substantially by removing any of the UGRS items. All subscales of the UGRS demonstrated good internal consistency: Reactions, α = .84, Injustice, α = .87, Counterfactuals, α = .88, Meaning, α = .88, and Relationships, α = .84. The item–total correlations of the UGRS items with the total score ranged from *r*_itc_ = .57 (item 1) to *r*_itc_ = .73 (item 15) (see Table 1). The mean item–total correlation was *r*_itc_ = .63. The mean inter-item correlation was *r* = .44, range *r* = .18 - .79. Mean inter-item correlation was highest between items 1 and 2, *r* = .79, and lowest between 1 and 7, *r* = .18.

**Table 1:** Reliability of the Swedish Utrecht Grief Rumination Scale.

Item	*Mean*	*SD*	Corrected item-total correlation *r*_itc_	Cronbach's alpha if item is deleted
1	2.99	1.21	.57	.92
2	2.83	1.25	.66	.92
3	1.94	1.21	.59	.92
4	2.16	1.39	.63	.92
5	2.55	1.48	.64	.92
6	2.54	1.28	.71	.91
7	2.11	1.22	.60	.92
8	2.39	1.42	.66	.92
9	1.91	1.18	.58	.92
10	1.90	1.34	.61	.92
11	2.27	1.38	.64	.92
12	2.99	1.45	.68	.91
13	2.55	1.35	.58	.92
14	2.15	1.23	.62	.92
15	3.24	1.29	.73	.91

### Construct validity

#### Factor structure

**Fig 1:**
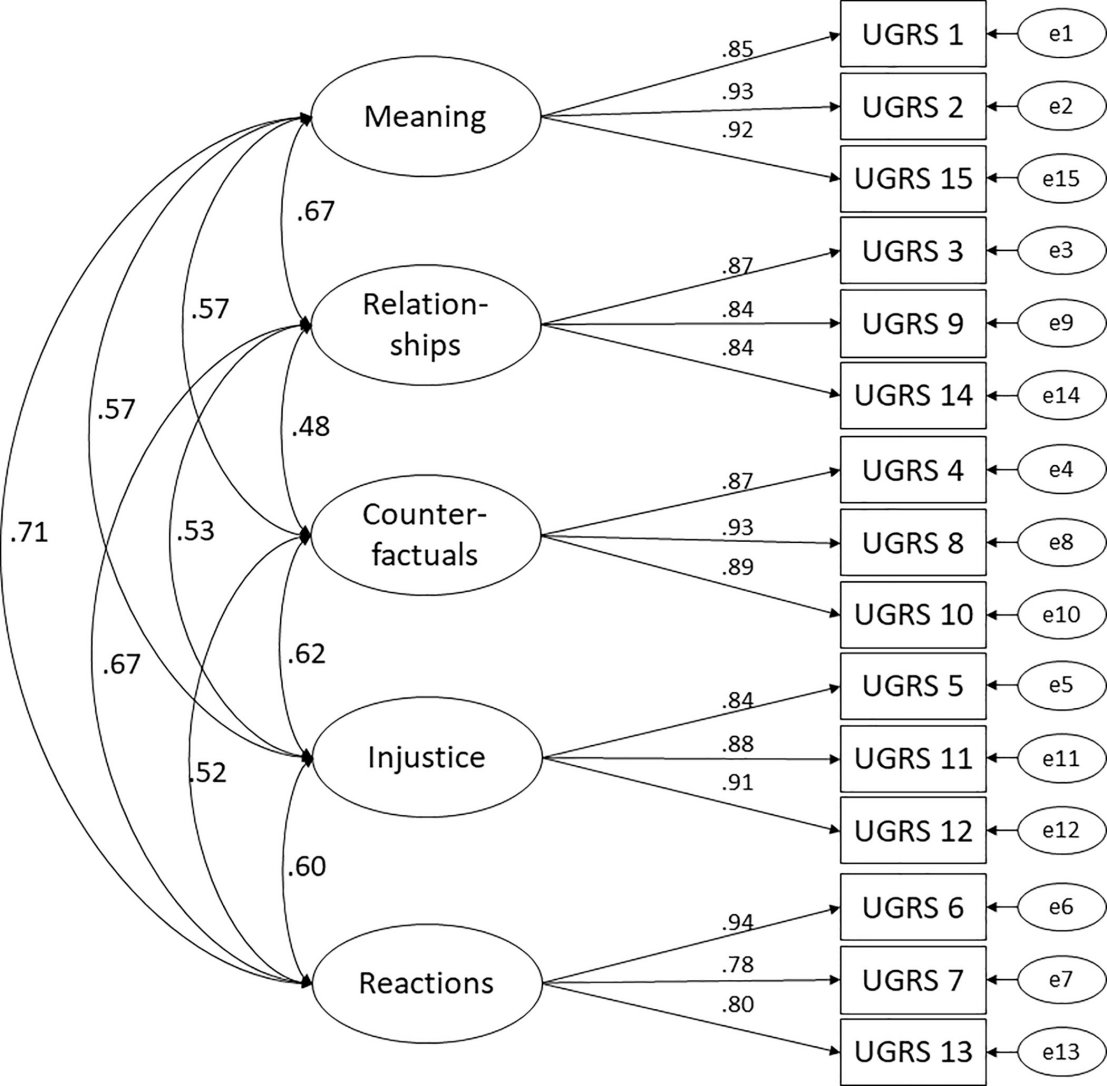
Correlated five-factor structure of the Swedish Utrecht Grief Rumination Scale. Error terms are denoted with a small ‘e’. All path coefficients are significant at p < .001.

**Fig 2:**
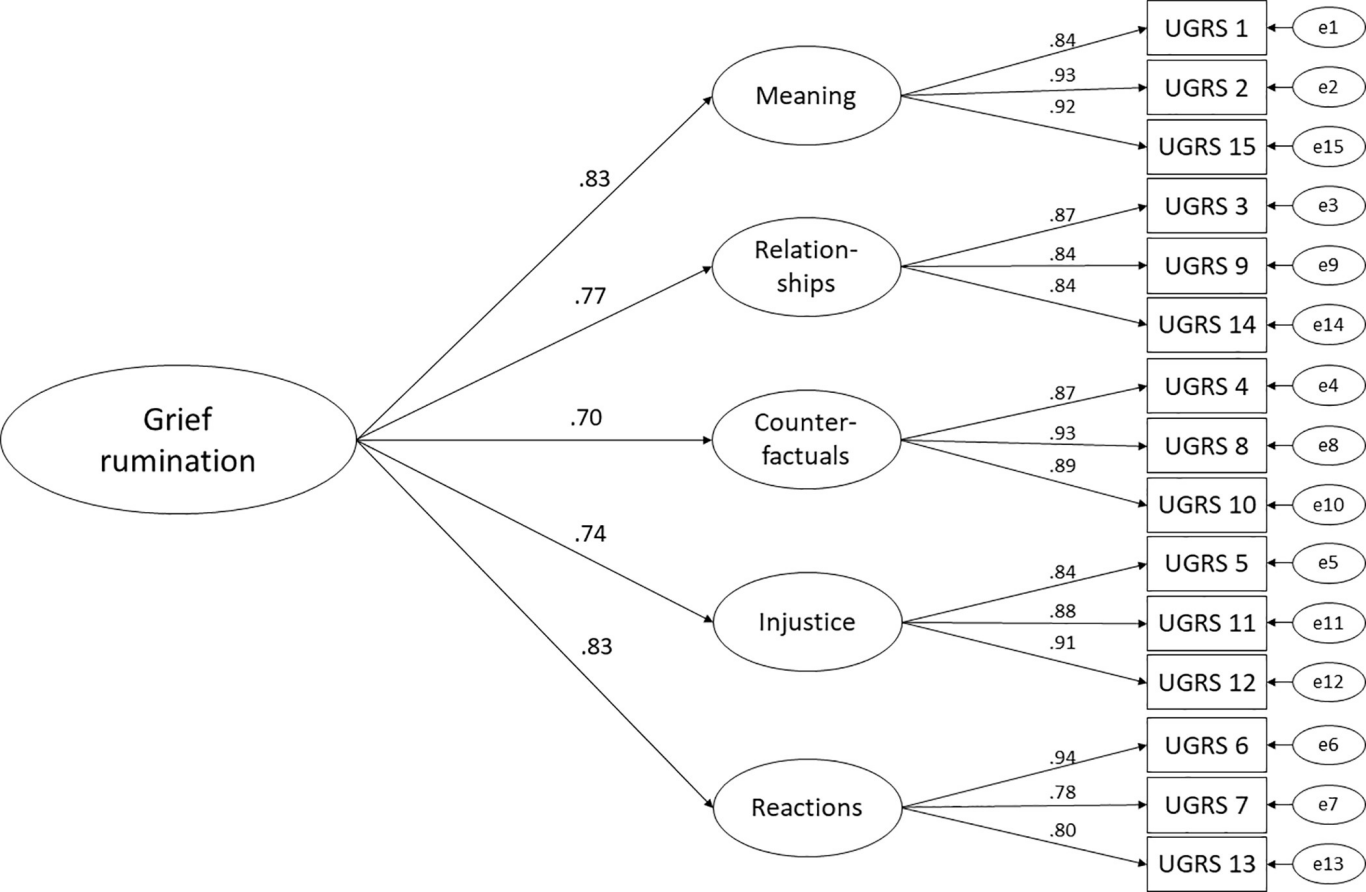
Second-order five-factor structure of the Swedish Utrecht Grief Rumination Scale. Error terms are denoted with a small ‘e’. All path coefficients are significant at p < .001.

Based on previous research [23, 31], using confirmatory factor analyses, two models were tested, a five-factor correlated model (Fig 1 for the path diagram) and a second-order five-factor model in which the five subscales are themselves the indicators for one higher-order factor, i.e., grief rumination (Fig 2 for the path diagram). The models fit indices are presented in Table 2 and both models yielded an acceptable model fit, however, the hierarchical model provide a marginally better fit.

**Table 2:** Fit indices for the confirmatory factor analyses on correlated and hierarchical factor models for the Swedish UGRS.

Model	χ^2^	*df*	χ^2^*/df*	*p*	CFI	TLI	RMSEA [90% CI]	WRMR
Five- factor	255.864	80	3.20	< .001	0.968	0.959	0.099 (.085; .112)	.933
Second-order five-factor	237.641	85	2.80	< .001	0.973	0.966	0.089 (.076; .103)	1.046

*Note*. CFI: Comparative Fit Index; TLI: Tucker-Lewis Index; RMSEA: Root mean square error of approximation; WRMR: Weighted root-mean-square residual.

#### Correlations among UGRS subscales

The correlations between the UGRS subscales and the total sum scores where all high, whereas the UGRS subscale inter-correlations ranged between .41 and .56, which indicates that the subscales tap into a similar construct yet are not interchangeable (Table 3).

**Table 3:** Inter-correlations among the Utrecht Grief Rumination Scale (UGRS) total and subscale scores, and correlations between UGRS scores and other constructs.

	UGRS total	Meaning	Relation-ships	Counter-factuals	Injustice	Reactions
UGRS total	-					
UGRS subscales:						
Meaning	.79***	-				
Relationships	.75***	.54***	-			
Counterfactuals	.77***	.49***	.41***	-		
Injustice	.80***	.50***	.47***	.58***	-	
Reactions	.79***	.56***	.55***	.48***	.53***	-
PG-13	.71***	.62***	.50***	.57***	.54***	.51***
PCL-5	.71***	.55**	.51***	.59***	.52***	.59***
MADRS	.52***	.46***	.45***	.43***	.31***	.41***
GAD	.54***	.44***	.44***	.46***	.39***	.40***
ISI	.41***	.35***	.26***	.34***	.30***	.35***
Parents gender	.30***	.31***	.20**	.21**	.28***	.18**
Child gender	.06	.06	.05	.03	.04	.03
Child age at death	.02	.08	-.16**	.09	.02	.05
Time since loss	-.11	-.13*	-.10	-.07	-.01	-.10

*Note*: PG-13: Prolonged Grief-13; PCL-5: Posttraumatic Stress Disorder Checklist for DSM-5; MADRS: Montgomery-Åsberg Depression Rating Scale; GAD-7:The Generalized Anxiety Disorder scale, ISI: Insomnia Severity Index; Gender variables were dummy-coded: 0 for female and 1 for male.

* *p* < .05

** *p* < .01

*** *p* < .001; Bonferroni corrected threshold: *p* < .0013 (all *p* with *** are significant after Bonferroni correction).

#### Discriminant validity

Discriminant validity of the UGRS was examined by dividing the sample into a group with and a group without a possible diagnosis of PGD. The UGRS total scores and subscales differed significantly between both groups, with relatively higher UGRS total and subscale scores in the possible PGD group (see Table 4).

**Table 4:** Differences in rumination levels between individuals with a possible prolonged grief disorder and those without.

	Possible PGD (*n* = 74)		No possible PGD (*n* = 150)				
	*Mean*	*SD*	*Mean*	*SD*	*t*	*df*	Cohen’s *d*
UGRS total	48.27	12.34	30.63	9.86	10.72***	120	1.64
Meaning	11.61	2.89	7.75	2.79	9.61***	222	1.37
Relationships	7.86	3.65	5.05	2.39	6.02***	105	0.98
Counterfactuals	9.16	3.91	5.08	2.75	8.06***	110	1.28
Injustice	10.24	4.12	6.63	3.03	6.70***	113	1.05
Reactions	9.39	3.40	6.11	2.77	7.20***	122	1.10

*Note*: PGD: Prolonged Grief Disorder.

*** *p* < .001

#### Concurrent validity

In support of concurrent validity of the UGRS, there were significant positive correlations between UGRS scores and all concurrently assessed psychopathology symptoms, i.e., prolonged grief, posttraumatic stress, depression, anxiety and insomnia (see Table 3). The UGRS total had significantly stronger correlations with prolonged grief, *r* = .71, and posttraumatic stress symptoms, *r* = .71, than with depression, *r* = .52; *z*(226) = 3.28, *p* < .001, anxiety, *r* = .54; z(226) = 2.99, *p* < .01, and insomnia symptoms, *r* = 41; *z*(226) = 4.77, *p* < .001.

A hierarchical multiple regression of background characteristics, symptoms of psychopathology, and rumination levels on symptoms of prolonged grief (Table 5). In a first step, sociodemographic and loss-related variables that significantly correlated with prolonged grief symptoms (see Table 3) were entered in the hierarchical model. Parents gender and age of the child at time of death were significantly associated with prolonged grief symptoms in this sample, *F*(3, 212) = 5.70; *p* = .001, *adj*.*R*^*2*^ = .06. In the next step, measures of psychopathology (i.e. depression, anxiety, posttraumatic stress, and insomnia) were entered. In this step, both symptoms of depression and posttraumatic stress, as well as time since loss, contributed significantly to the prediction of prolonged grief symptoms, *F*(7, 208) = 73.01; *p* < .001, *adj*.*R*^*2*^ = .70. In the last step, UGRS scores were included in the model and improved the prediction of prolonged grief symptoms significantly, *F*(1, 207) = 76.85; *p* < .001, *adj*.*R*^*2*^ = .74.

**Table 5:** Multiple hierarchical regression analysis.

	B	SE B	β	*ΔR*^*2*^
Step 1 (*df* = 3, 212)				.075**
Constant	31.85	2.06	-	
Gender parent	-3.14	1.26	-.16*	
Age child at death	.28	.10	-.18**	
Time since loss	-.89	.44	-.14*	
Step 2 *(df* = 4, 208)				.636***
Constant	21.28	1.27	-	
Gender parent	-.54	.74	-.28	
Age child at death	.11	.06	.07	
Time since loss	-.86	.25	-.13**	
Posttraumatic stress	.33	.04	.56***	
Depression	.32	.08	.31***	
Anxiety	.08	.10	.05	
Insomnia	-.08	.07	-.06	
Step 3 (*df* = 1, 207)				.037***
Constant	14.58	1.70	-	
Gender parent	.50	.72	.03	
Age child at death	.14	.06	.09*	
Time since loss	-.62	.24	-.09*	
Posttraumatic stress	.20	.04	.34***	
Depression	.36	.07	.35***	
Anxiety	.05	.10	.03	
Insomnia	-.09	.07	-.07	
UGRS	0.21	0.04	.29***	

*Note*: Results of the multiple hierarchical regression with forced blockwise entry. Criterion: prolonged grief symptoms (PG-13 score). Demographic characteristics and time since loss entered in the first block, posttraumatic, depressive, anxiety and insomnia symptoms in the second, and grief-related rumination (UGRS Score) in the last block. Sex was dummy-coded: 0 for female and 1 for male.

* p < .05

** *p* < .01

*** *p* < .001.

## Discussion

This is the first study to validate a Swedish version of the Utrecht Grief Rumination Scale (UGRS). The analyses of reliability and validity indicates that the Swedish UGRS has satisfactory psychometric properties in parents who have lost a child to cancer.

The reliability of the UGRS subscales and the total UGRS were supported by acceptable Cronbach’s alpha values, inter-item correlations, item-total correlations and mean inter-item correlations. The Cronbach’s alpha and item-total correlations were comparable with those found in previous validation studies of the English and the Dutch UGRS [23] and the German UGRS [32]. For example, the Cronbach’s alpha of the whole scale in the present study was .92 and was .89 in the German UGRS validation study.

The results supported the construct validity of the UGRS. Based on previous research two confirmatory factor analyses were conducted to test a five-factor correlated model and a second-order five-factor model. The models differed only marginally and both showed an acceptable model fit on nearly all fit indices (CFI, TLI, WRMR), but the second-order five-factor model had a slightly better fit. It should briefly be noted that the RMSEA was the only fit index for which our model did not reach the standard for acceptable fit (.08) advocated by Kline [46]. However, cut-offs for acceptable fit for the RMSEA have ranged from .05-.10, and statisticians have warned about rigid use of RMSEA cut-offs [49]. One particular concern is that the RMSEA is sensitive to small sample sizes and mild model misspecification. Taking such considerations (and our relatively small sample) into account, the second order five-factor model appears to be the preferred model for the Swedish UGRS data over the five-factor correlated model.

These results are comparable with the results of a study of Eisma and colleagues [23] which demonstrated that the two models had very similar fit indices, however, the five-factor correlated model performed marginally better in both the Dutch and the English sample. The German UGRS [32] factorial analyses mirror the results of Eisma and colleagues [23]. Taken together, these results support that the total UGRS is a general measure of grief-specific rumination, yet that the subscales each represent different aspects of grief rumination. So, the UGRS can be used to assess subtypes of grief rumination, and adding scores from the subscales forms a total score that represents general grief rumination. This is further supported by the finding that the Swedish UGRS item inter-correlations were predominantly moderate to high and the subscale inter-correlations were moderate, which indicates that the items tap into a similar construct but are not interchangeable.

Discriminant validity of UGRS was examined by comparing participants with and without a possible PGD diagnosis on their UGRS total and subscale scores. The group with a possible PGD diagnosis had significantly higher scores on grief rumination and grief rumination subtypes than those without. The total score was elevated strongly in the group with possible PGD when compared with the group without, Cohen’s *d* was 1.47, which is similar to the results found in Doering et al. [32], with a Cohen’s *d* of 1.66.

There was a strong correlation between grief rumination and prolonged grief symptoms, which replicate findings from previous studies [23, 32]. Grief rumination was supported as it predicted prolonged grief symptoms over and above relevant background variables and symptoms of posttraumatic stress, depression, anxiety and insomnia, that is, even after accounting for variation in symptom severity of various types of psychopathology, those with greater rumination evidenced more severe prolonged grief symptoms. This is in line with the claim that grief rumination is a unique mechanism underlying disturbed grief responses. It has been suggested that rumination, as well as other forms of repetitive negative thinking, are trans-diagnostic risk factors for a variety of disorders (for a review, see [50]). The associations between grief rumination and symptoms of various other types of psychopathology found in this study seem to support this claim. Moreover, these findings align with other previous studies demonstrating that grief rumination is linked with different mental health problems following loss, including anxiety, depression and posttraumatic stress [18].

A notable finding was that the positive associations between rumination on the one hand and prolonged grief and posttraumatic stress symptoms on the other hand were not significantly different. Generally, associations between rumination and prolonged grief symptoms have appeared somewhat higher than those between rumination and posttraumatic stress symptoms in previous studies [31, 51]. While this result may seem unexpected, it can be explained. First, the proposed contents of rumination in PTSD and prolonged grief partially overlap (e.g., both contain repetitive counterfactual thoughts about the negative life-event and analyses of why the negative life-event occurred). Second, the cognitive avoidant function of rumination has been proposed to perpetuate both types of mental health problems in similar ways [24, 52]. As such, it may not be surprising that in a subgroup of bereaved persons at risk of developing PTSD and prolonged grief (i.e. bereaved parents), rumination is not differentially linked with these different types of mental health problems.

To the best of our knowledge, this was the first study to demonstrate a significant, moderate, positive association between grief rumination and sleep disturbance following bereavement. These findings are in line with a large body of research demonstrating an association between measures of rumination and worry and sleep problems (for a review: [29]). An influential cognitive model of insomnia by Harvey [53] holds that repetitive thinking about not sleeping enough and the impact of sleep disturbance leads to distress and arousal, which in turn leads to selective attention and monitoring of sleep-related threats. This results in an over-approximation of actual sleep problems and affect during daytime, which subsequently maintains repetitive thinking and raises arousal, causing sleep problems. Potentially, grief rumination may be interlinked with sleep-related repetitive thought, an thereby play a role in the perpetuation of sleep problems commonly experienced by bereaved people [54].

Some limitations of this study warrant mention. A limitation of the study is the cross-sectional design which prevents the demonstration of the temporal stability and predictive validity of the Swedish UGRS, which has been demonstrated in Dutch bereaved samples [31, 55, 56]. Hence, longitudinal research is needed to investigate the predictive value of the Swedish UGRS. Another limitation is that convergent validity was not investigated, thus more research is needed to test the association of the Swedish UGRS with other measures of rumination. A further limitation is that the results are based on self-report data, therefore certain response tendencies such as response bias and recency bias may have influenced study outcomes. Given the exclusive focus on bereaved parents in this study, findings may not be generalizable to other bereaved populations who have experienced other types of loss. However, the strong comparability of present findings with findings on psychometric evaluations of the UGRS in other languages, conducted in more heterogeneous samples, does suggest that the present scale could prove useful to study rumination in bereaved samples in general. Future research in larger, more heterogeneous Swedish bereaved samples can help establish if this is the case. In addition, given that the prolonged grief levels in the present sample ranged from non-clinical to clinical, further studies in clinical samples would be valuable.

## Conclusions

In summary, this study supports the use of the Swedish UGRS to measure grief rumination in bereaved parents. The Swedish UGRS was shown to have good reliability. The findings support the UGRS construct validity, discriminant validity, and concurrent validity. As such, UGRS appears a useful scale in research to increase the understanding of the role that grief rumination plays in mental health problems in bereaved parents in Sweden. Should future research on other samples of bereaved persons and clinical samples confirm the evidence for the validity of the Swedish UGRS, it may prove to be a useful instrument to study rumination in Swedish bereaved people in general.
